# A multidisciplinary weight management intervention for adults with severe mental illness in forensic psychiatric inpatient services (Motiv8): a single blind cluster-randomised wait-list controlled feasibility trial

**DOI:** 10.3389/fpsyt.2024.1457864

**Published:** 2024-11-18

**Authors:** Rebekah Carney, Heather Law, Hany El-Metaal, Mark Hann, Gemma Shields, Siobhan Savage, Ingrid Small, Richard Jones, David Shiers, Gillian Macafee, Sophie Parker

**Affiliations:** ^1^ Youth Mental Health Research Unit, Greater Manchester Mental Health NHS Foundation Trust, Manchester, United Kingdom; ^2^ Division of Psychology and Mental Health, University of Manchester, Manchester, United Kingdom; ^3^ Greater Manchester Mental Health NHS Foundation Trust, Manchester, United Kingdom; ^4^ Division of Population Health, Health Services Research, and Primary Care, School of Health Sciences, University of Manchester, Manchester, United Kingdom; ^5^ Psychosis Research Unit, Greater Manchester Mental Health NHS Trust, Manchester, United Kingdom; ^6^ School of Medicine, University of Keele, Staffordshire, United Kingdom

**Keywords:** secure services, forensic, physical health, multidisciplinary, lifestyle intervention, randomised controlled trial

## Abstract

**Background:**

People with severe mental illness experience physical health inequalities and a 15–20-year premature mortality rate. Forensic inpatients are particularly affected by restrictions on movement, long admissions, and obesogenic/sedative psychotropic medication. We aimed to establish the feasibility and acceptability of Motiv8, a multidisciplinary weight management intervention co-produced with service users for forensic inpatients.

**Methods:**

A randomised waitlist-controlled trial of Motiv8(+Treatment-As-Usual) vs.TAU was conducted in medium-secure forensic services in Greater Manchester. Motiv8 is a 9-week programme of exercise sessions, diet/cooking classes, psychology, physical health/sleep education, and peer support. Physical and mental health assessments were conducted at baseline/10-weeks/3-months. A nested qualitative study captured participant experiences. A staff sub-study explored ward environment.

**Results:**

We aimed to recruit 32 participants (four cohorts). The trial met recruitment targets (n=29, 90.9%; 4 cohorts, 100%), participants were randomised to Motiv8+TAU (n=12) or waitlist (control) (n=17). Acceptable retention rates were observed (93.1%, 10-weeks; 72.4%, 3-months), and participants engaged well with the intervention. The blind was maintained, and no safety concerns raised. Assessment completion was high suggesting acceptability (>90% for people retained and engaged in the study). Participants reported high levels of satisfaction.

**Conclusions:**

The trial was not powered to detect group differences. However, data suggests it is feasible to conduct a rigorous, methodologically robust study of Motiv8 vs.TAU for adults on forensic inpatient units. Motiv8 was acceptable with potential promise providing evidence to proceed to a definitive trial for males. A larger trial is needed to explore potential effectiveness and reduce physical health inequalities for people with SMI.

**Clinical trial registration:**

https://doi.org/10.1186/ISRCTN13539285, identifier ISRCTN13539285.

## 1 Introduction

People with Serious Mental Illness (SMI) experience physical health inequalities and a 15-20-year loss of life (1–3). This has been labelled a ‘national scandal’, leading to increased calls to action, such as the Lancet commission for physical healthcare, updated guidance from National Health Service England (top 10 priorities to improve the physical health of people living with SMI) and World Health Organization (WHO) recommendations (4–8). The Office for Health Improvement and Disparities (2023) published updated prevalence rates showing compared with all patients, those with SMI have higher prevalence of obesity, asthma, diabetes, chronic obstructive pulmonary disease (COPD), coronary heart disease (CHD), stroke, hypertension and cancer. Individuals in forensic services are particularly vulnerable. Forensic psychiatric services have a dual purpose; to treat people who pose a risk to themselves or others and address offending behaviours. Approximately, 8000 people reside in forensic psychiatric services in the UK (9). Admissions usually exceed 5 years, and over 15 years for 20% of people (6, 10–12). Forensic psychiatric services receive a quarter of the mental health funding budget (13, 14), and physical comorbidities are a key predictor of total healthcare costs (15).

Obesity rates in forensic psychiatric services can reach 70%, and correlate with length of stay (16, 17). Cardiovascular disease and type 2 diabetes are more prevalent on secure units than generic inpatient units (16, 18, 19). People in forensic psychiatric services are more susceptible to risk-taking behaviours associated with high rates of adverse childhood experiences (20, 21). There are high rates of engagement with adverse health behaviours (e.g. sedentary activity) and polypharmacy of obesogenic and sedative psychotropic medication is common (22, 23). The ‘obesogenic’ nature of the inpatient environment also affords fewer opportunities to be active due to restrictions on movement, reduced access to outdoor spaces, and increased access to unhealthy foods (24).

WHO recommend increasing physical activity, reducing sedentary behaviour, and improving lifestyle to improve cardiometabolic health for people with SMI (25). Despite evidence showing physical health interventions benefit mental and physical health, well conducted studies in forensic settings are limited (26–30). An NHS-commissioned review identified only one weight management randomised controlled trial (RCT), along with several small uncontrolled studies (31, 32). Existing interventions often fail to include control groups, standardised outcome measures, and long-term follow-ups. There is often limited input from service users in intervention development, underrepresenting the ‘patient voice’, yet co-production is vital to increase sustainability and improve engagement (33).

### 1.1 Aims and objectives

We aim to address this evidence gap and explore the feasibility of Motiv8. Motiv8 is a 9-week multidisciplinary intervention which was co-developed, co-produced and co-facilitated with service users to improve cardiovascular health of people on forensic psychiatric units, (see (34) for further details). The primary aim is to conduct a randomised waitlist-controlled feasibility trial of Motiv8 vs.TAU for adults on forensic mental health units, to investigate the acceptability, feasibility, and potential effectiveness of Motiv8 to supplement standard care.

## 2 Methods

### 2.1 Trial design

We conducted a prospective, single blind, cluster-randomised controlled feasibility trial with two conditions; Motiv8+treatment as usual (TAU), versus TAU+waitlist control (with Motiv8 delivered after TAU) (see **Supplementary Materials** for flow chart). The study took place in adult medium secure forensic mental health inpatient services at Greater Manchester Mental Health NHS Foundation Trust (GMMH NHS FT). All authors assert that all procedures contributing to this work comply with ethical standards of the relevant national and institutional committees on human experimentation and with the Helsinki Declaration of 1975, as revised in 2008. All procedures involving human subjects were approved by the Health Research Authority (HRA) London Bromley Research Ethics Committee [25^th^ October 2021, 21/LO/0658, IRAS 299909]. It was prospectively registered on the ISRCTN registry [ISRCTN13539285]. The study protocol was published prior to study end (34). The trial was conducted and reported in line with the CONSORT extension to RCTs (35). An independent “Experts by Experience” group was established prior to study set-up and provided study oversight.

### 2.2 Participants

Participants were recruited from medium secure units at forensic services at GMMH NHS FT and were eligible if they met the following inclusion criteria.

#### 2.2.1 Inclusion

- Adult inpatient (18+) of a medium secure unit at GMMH NHS FT.
- Mental health diagnosis requiring treatment from forensic psychiatric services.
- Capacity to provide informed consent.
- Physically able and medically safe to exercise (according to the Physical Activity Readiness Questionnaire).

#### 2.2.2 Exclusion

- Inability to provide informed consent in line with ethical requirements.
- Previous Motiv8 participant.
- Insufficient command of English/communication difficulties preventing engagement in written informed consent, validity of research assessments or understanding of the programme.

Clinical leads of forensic services were consulted to identify potential wards. The research team liaised with clinical teams on wards to inform them of the study and provide details about inclusion criteria. Clinical teams approached patients to see if they were interested and obtain consent-to-contact. Researchers then arranged to meet with potential participants on the ward to discuss the study. A member of the research team had approvals to screen patient lists and identify potentially eligible participants.

All participants provided written informed consent prior to undertaking research procedures and completed a physical health risk assessment prior to engaging in Motiv8. We aimed to recruit 32 participants making up four cohorts, with a maximum of eight participants per cohort. This was due to pragmatic limitations associated with the need to keep groups small due to complex needs of service users requiring a set staff-to-patient ratio, and time/funding constraints. We aimed to recruit cohorts on a ward-by-ward basis, a decision based on previous consultations with people with lived experience as it was believed to avoid conflict between wards and meet COVID restrictions which prevented wards mixing. Eligible wards were required to have up to eight potential participants. This was not feasible for one cohort. Therefore, through discussions with the experts-by-experience group and service leads at the trust, participants from two wards were combined.

### 2.3 Randomisation

Participants were cluster randomised by cohort using the free web-based system (Sealed Envelope™, www.sealedenvelope.com) by a research administrator. Allocation was communicated to the chief investigator, study management, facilitators, and care teams of participating wards. Research assistants, the statistician and health economist remained blinded. Participants were informed of their randomisation outcome by letters sent to the wards, and clinicians who were informed by the administrator. Blinding remained in place until all outcome measures were collected and analysed. Measures to maintain blindness included separate offices and workspaces for facilitators and researchers, protocols for answering phones, secretarial support and separate secure drives to store password protected documents. The blind was successfully held.

### 2.4 Procedures

Recruitment occurred at two timepoints (Dec 2021/July 2022) and two cohorts were recruited at each timepoint. Cohorts were randomised to receive Motiv8 straight away or placed on a waitlist to receive Motiv8 after the first follow-up timepoint. Motiv8 was provided along with TAU which was the usual provision of inpatient care for people with SMI and remained unchanged throughout the study. Assessments were conducted by trained, blinded researchers at baseline (pre-Motiv8/TAU), 10-weeks (post-Motiv8/TAU), and after another 12-weeks (TAU/waitlist Motiv8 (**Supplementary Data Sheet 1**). Demographics and clinical data were collected via self-report measures and researcher administered questionnaires.

### 2.5 Intervention

Motiv8 is a 9-week intensive programme co-developed with service users to improve the cardiovascular and metabolic health of people on forensic inpatient units. It was developed with service users who were inpatients at the hospital and clinical teams. It aims to increase activity levels, improve diet, and use psychological guidance to maintain good physical health using goal-based techniques. It was delivered in groups to each cohort consecutively (the waitlist design meant all participants were offered Motiv8). Sessions took place in clinical areas in inpatient NHS facilities (e.g., ward or recovery academy kitchen, sports hall, meeting rooms and therapy rooms).

**Figure 1:**
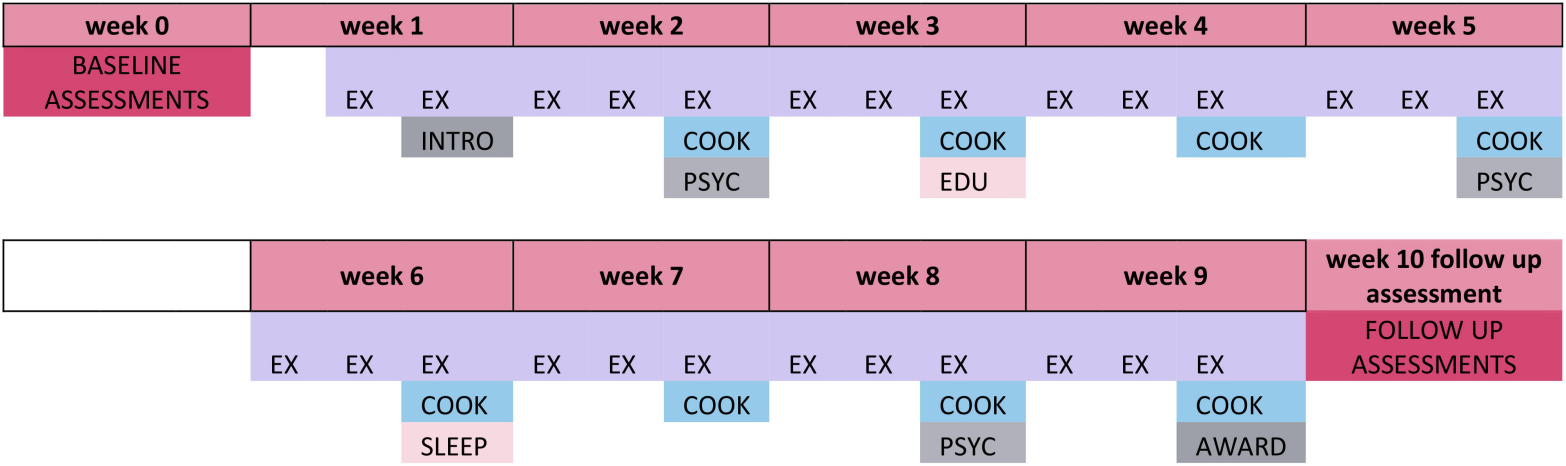
Example schedule for Motiv8 intervention. INTRO, Introduction; EX, Exercise Sessions; COOK, Nutrition Sessions; EDU, Physical Health Education; PSYC, Psychology Sessions; SLEEP, Sleep Session; AWARD, Awards Session.

Motiv8 is multidisciplinary including several components to support physical health: exercise sessions, cooking/nutrition classes, physical health education, psychology sessions, sleep education, peer support and a medication review (See **Figure 1**; **Table 1** for an example schedule). Motiv8 was facilitated and delivered by experienced occupational therapists, dietitians, psychologists, pharmacists, physicians, exercise and sport recreation workers, nurses, and peer mentors. Weekly supervisions were conducted internally, and intervention fidelity was monitored through regular meetings and paperwork. A person with lived experience co-facilitated and co-delivered sessions and provided peer support. An intervention booklet was created by the experts-by-experience group and research team which consisted of resources, activities and prompts for goal setting/review of progress. To increase morale, emphasis was placed on achievements and community, and participants attended an awards ceremony upon completion where they received a trophy, certificate, Motiv8 t-shirt, Motiv8 water bottle and voucher. Findings from successful pilot work across five cohorts (n=32) enabled Motiv8 to be iteratively updated with service user input and suggested that it may be feasible and beneficial for participants. [See https://doi.org/10.21203/rs.3.rs-3087194/v1 (34)].

**Table 1:** Example schedule for Motiv8 intervention.

	Exercise	Diet	Psychology group	Other
**Week 1**	2 x sessions			Introduction to Motiv8
**Week 2**	3 x sessions	1 x session		
**Week 3**	3 x sessions	1 x session	1 x session	Physical health education session
**Week 4**	3 x sessions	1 x session		Sleep session
**Week 5**	3 x sessions	1 x session	1 x session	
**Week 6**	3 x sessions	1 x session		Pharmacy review
**Week 7**	3 x sessions	1 x session	1 x session	
**Week 8**	3 x sessions	1 x session		
**Week 9**	3 x sessions	1 x session		Award ceremony

### 2.6 Outcomes

The primary aim was to assess the acceptability and feasibility of the trial and associated processes including the intervention and assessments. Key markers of feasibility included recruitment rates, follow-up retention rates, completion of clinical outcomes, and safety. Intervention acceptability was assessed via receiving a dose of the intervention, attendance, and adherence to the intervention, as well as subjective participant experiences and feedback from qualitative interviews. Participant interviews were analysed using in-depth qualitative methods and will be reported separately to provide a comprehensive, complete, and transparent account of our findings. The proposed primary outcome for a definitive trial was change in weight at 10-weeks/3-months. The study was not powered sufficiently to detect significant differences between groups and primary outcomes were to establish feasibility.

Clinical outcomes included a purpose-built form to collect basic sociodemographic information (e.g. ethnicity, gender, education status) and clinically relevant information (diagnoses, admission history, inpatient status, physical health conditions). Physical health measures included BMI, resting blood pressure, pulse rate, hip/waist/neck/chest circumference collected using disposable tape measures and recorded on a purpose-built questionnaire. To estimate cardiovascular fitness the six-minute walk (36) and standing jump test (37) were completed. Mental health outcomes included wellbeing [Warwick Edinburgh Mental Wellbeing Scale, WEMWEBS (38)], symptoms of depression and anxiety [Hospital Anxiety and Depression Scale (39)] and negative symptoms [Scale of Negative Symptoms, SNS (40)]. Behavioural outcomes included physical and sedentary activity [Simple Physical Activity Questionnaire, SIMPAQ, (41)]; dietary intake (24-hour diet recall including time of consumption/portion size, (42); sleep quality and quantity [PROMIS Sleep Disturbance short form 8-item and PROMIS Sleep-Related Impairment short form 4-item (43)]; and smoking habits using a purpose-built demographics questionnaire developed from existing measures. Functioning was assessed through occupational therapy [Model of Human Occupational Screening Tool, (44)]. Outcomes to inform cost-effective analysis for a future study included health status [EQ-5D-5L, (45)], quality of life (Recovering Quality of Life, ReQoL (46), and medication side effects [Liverpool University Neuroleptic Side Effect Rating Scale, LUNSERS, (47)].

### 2.7 Statistical methods

This feasibility study is not powered to test for intervention effectiveness therefore, our analyses are descriptive. The primary focus is on summaries of key indicators of success of the study (e.g. rates of recruitment, engagement, retention, and satisfaction). Following intention-to-treat principles, ‘logistics’ data is reported according to the CONSORT extension to RCTs (48) including: the number of prospective participants who were approached, subsequently deemed eligible and consented; the number of participants completing baseline questionnaires and who were randomised (by cohort); the number of participants who received their intended intervention and who were assessed at follow-up (including any reasons for loss to follow-up); the number of participants providing ‘complete’ clinical outcome data at each assessment.

Descriptive summaries of baseline demographic data are reported. For the latter, we present the median, the inter-quartile range and the data range due to the small numbers and likely skewness of each measure. We also present descriptive data on change in outcomes between baseline and week 10 for weight, WEMWBS and other clinical indicators of interest. As Motiv8 was delivered in cohorts, intra-cohort correlation will be present in the outcomes. A sample size calculation for a definitive trial will require an estimate of the intra-cohort correlation. Although such estimates have been calculated, the number of cohorts is likely to be too small here for them to be accurate.

### 2.8 Role of the funding source

This work was funded by the NIHR via the Research for Patient Benefit Programme (Grant Reference Number: RfPB NIHR201482). The funder had no input to the study design, delivery or interpretation of results, and the views expressed here are that of the authors.

## 3 Results

### 3.1 Participant flow

**Figure 2:**
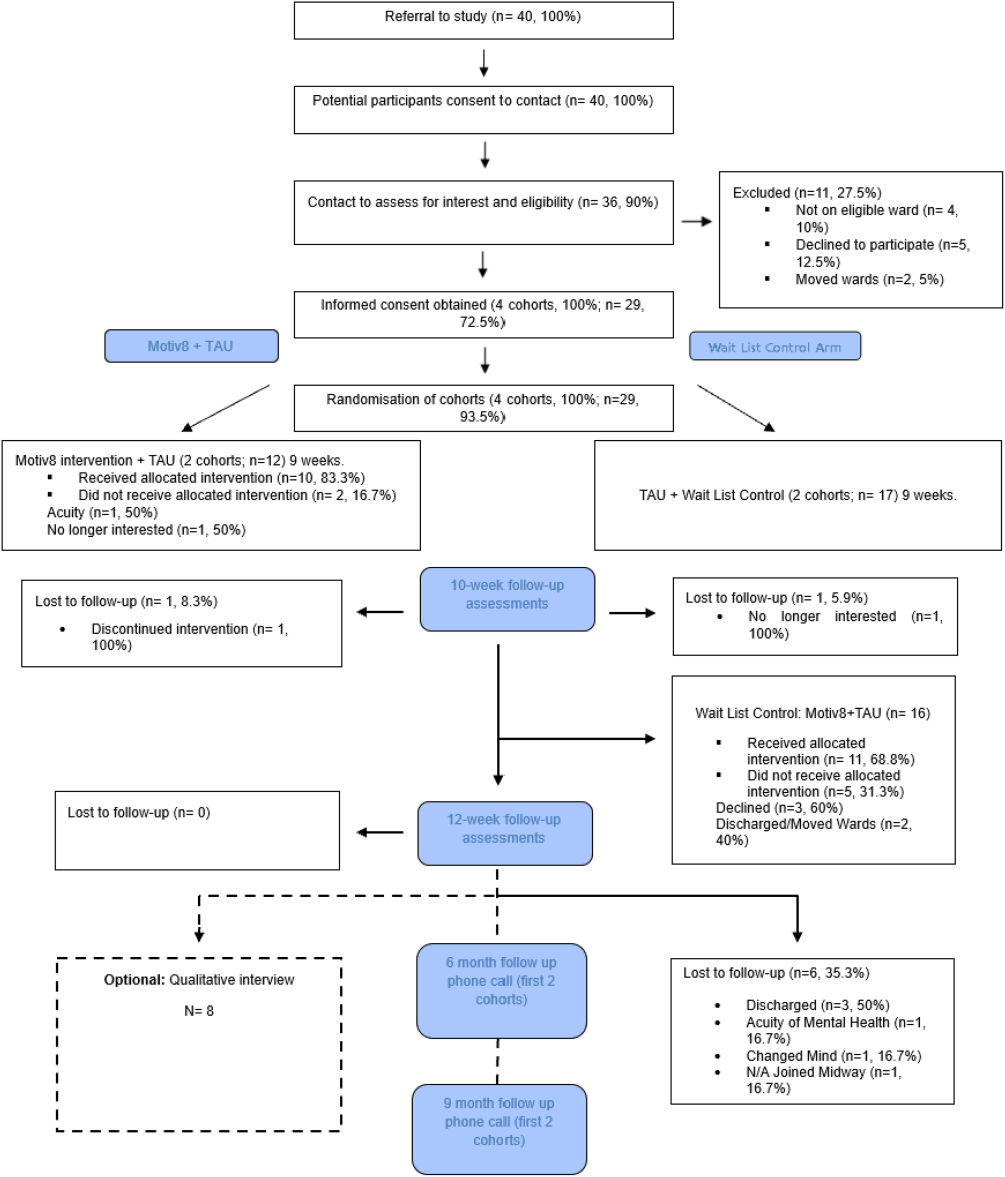
CONSORT participant flow digram.

Participants were recruited at two timepoints, December 2021/July 2022 and final follow up assessments were completed in December 2022. Four cohorts were successfully recruited (100% target) consisting of 29 participants (90.6% target). Forty potential participants were referred and a referral recruitment rate of 1.4:1 was observed (n=40 referrals). All referrals were screened, six were ineligible at referral (n=4, 10% not on eligible ward, n=2, 5% discharged before approached) and five declined when approached (12.5% referrals). 100% consented participants were randomised to either Motiv8 (n=12, 41.4%) or TAU+Waitlist Motiv8 (n=17, 58.6%). Participants were recruited from five medium secure treatment wards (4 male wards, 1 female ward). See **Figure 2** for consort.

Retention to the trial was 93.1% (n=27) at 10-weeks and 72.4% (n=21) at 3-months which almost fulfilled progression criteria. The main reason for loss-to-follow up was driven by participants being discharged or moved to another trust (n=5, 17.2%). Lower retention rate at 3-months may have been caused by an unprecedented incident which occurred in the latter stage of the trial resulting in high staff turnover and rapid patient discharge. Additionally, retention was significantly lower in Cohort 3 and 4 (83.3%, n=10, 10-weeks; 58.3%, n=7, 3-months), compared with Cohort 1and 2 (100%, n=17, 10-weeks; 82.4%, n=14, 3-months). After six-months, contact was attempted with the first two cohorts, 11 participants were contacted (64.7%) and almost all said they would take part in assessments in a definitive trial (91%). Completion of the proposed primary outcome (weight) was high 96.6% (n=28, 10-weeks) and 95.2% (n=20, 3-months). High levels of acceptability were found for all measures (completion rate 93.3%-95.7%, 3-months). Retention rate per cohort is included in **Supplementary Data Sheet 1**.

### 3.2 Baseline data

The socio-demographics of participants are as follows. Participants were predominantly male (89.7%, n=25), White-British/White Other (75.9%, n=22), single (93.1%, n=27) and over half had been an inpatient for over five years (55.2%, n=16). See **Table 2**. Almost all participants (n=28, 96.5%) had a diagnosis of a schizophrenia spectrum/psychosis related disorder and all were receiving medication, (93% antipsychotics, n=27). At baseline 85.7% (n=24) of participants were overweight or obese (median BMI 34.4, range 20.4-56.8). See **Table 3**. Full baseline characteristics will be published in detail elsewhere for completeness.

**Table 2:** Socioeconomic Demographics.

Variable of Interest	Total N	Average (s.d.)	Range
**Age at entry (years)**	29	36.5 (9.9)	20-61
Gender	Total N	N (yes)	%
Male	29	25	89.7%
Ethnicity			
White (British/Other)	29	22	75.9%
Mixed or Multiple Ethnic Group	29	3	10.4%
Black, Black British, Caribbean, or African	29	3	10.4%
Other	29	1	3.5%
Educational Status			
Higher/Further Education (University, A-Level, General Notational Vocational Qualification, Business and Technology Education Council)	29	6	20.7%
General Certificate Secondary Education or Equivalent	29	5	17.2%
Other^	29	5	17.2%
Reported no completed education	29	13	44.8%
Living Status Prior to Admission			
Hospital inpatient longer than 5 years	29	16	55.2%
With parents or other family	29	5	17.2%
Lives alone	29	2	6.9%
No fixed abode	29	2	6.9%
Other (justice system)	29	4	13.8%
Religious Beliefs			
No Religion	29	14	41.4%
Christian	29	9	31%
Other~	29	5	17.2%
Prefer not to say	29	1	3.5%
Legal Status*			
1983 MHA Section 37	29	6	20.7%
1983 MHA Section 37/41	29	10	34.5%
1983 MHA Section 47	29	1	3.5%
1983 MHA Section 47/49	29	5	17.2
1983 MHA Section 3	29	7	24.1%
Marital Status			
Single/Divorced	29	28	96.6%

^included prison courses, vocational (city and guilds).

~included Muslim, apostle, spiritual, unsure.

*see **Supplementary Data Sheet 1** for definitions of legal status.

**Table 3:** Socioeconomic demographics.

Variable of Interest	Total N	N	%		
Mental Health Diagnosis					
Paranoid Schizophrenia	29	21	72.4%		
Schizophrenia Unspecified	29	2	6.9%		
Schizoaffective Disorder	29	4	13.8%		
Delusional Disorder	29	1	3.5%		
Emotionally Unstable Personality Disorder	29	1	3.5%		
Previous Admissions					
At GMMH Secure Services	29	14	42.3%		
Any Other Services	29	17	58.6%		
Medication Class					
Antipsychotics	29	27	93%		
Antidepressants	29	16	55%		
Anxiolytics	29	8	28%		
Other Health Medication	29	24	83%		
Digestion Medication	29	14	48%		
Asthma Medication	29	8	28%		
Supplements (e.g. vitamins)	29	23	79%		
Any Other Medication	29	20	69%		
				**Median**	**Range**
Number of different mental health medications	29			3	1 to 6
Number of different mental health medications which have an impact on weight	29			2	0 to 6
		**N**	**%**		
Family History of Mental Health Conditions					
Mother	29	8	28%		
Father	29	4	14%		
Sister	29	6	21%		
Brother	29	4	14%		
Other (child, grandparent, uncle)	29	5	17%		
**Treatment Received**		**Current**		**Previous**	
		**N**	**%**	**N**	**%**
**Psychological Therapies (Total)**	29	**12**	**41.4%**	**27**	**93.1%**
Cognitive Behavioural Therapy (CBT)	29	4	14%	10	34%
Dialectical Behavioural Therapy (DBT)	29	1	3%	2	7%
Group Therapy	29	–	–	10	34%
Psychology Sessions	29	4	14%	–	–
Compassion Therapy	29	2	7%	1	3%
Psychological Schema Therapy	29	1	3%	1	3%
Exposure Response Therapy	29	–	–	1	3%
Psychosocial Therapy	29	–	–	1	3%
Cognitive Analytic Therapy	29	–	–	1	3%
**Offender Related (Total)**	29	**2**	**7%**	**5**	**17.2%**
Relapse Prevention	29	1	3%	3	10%
Psychology Offender Related Therapy	29	1	3%	–	–
Life Minus Violence	29	–	–	2	7%
**Other (Total)**	29	**1**	**3%**	**7**	**24.1%**
Family Therapy	29	–	–	–	–
Eye Movement Desensitisation and Reprocessing (EMDR)	29	–	–	1	3%
Art Therapy	29	1	3%	1	3%
Healthy Relationships	29	–	–	1	3%
Anger Management	29	–	–	1	3%
Speech Therapy	29	–	–	1	3%
Cognitive Skills/Advance Thinking	29	–	–	2	7%
**Substance Misuse**	29	**2**	**7%**	**1**	**3%**
Drug and Alcohol Counselling	29	2	7%	1	3%
Physical Health Indicators					
Recorded Medical Conditions^					
Asthma	29	7	24%		
T2 Diabetes	29	7	24%		
High Cholesterol	29	7	24%		
Hypertension	29	7	24%		
Epilepsy	29	2	7%		
Heart Arrythmia	29	2	7%		
Obstructive Sleep Apnoea	29	2	7%		
BMI					
Normal Weight (18.5-24.9)	28	4	14.3%		
Overweight (25-29.9)	28	7	25%		
Obese (30+)	28	17	60.7%		
Overweight or Obese (25+)	28	24	85.7%		

^Where N>1 per condition.

Clinical Demographics.

### 3.3 Numbers analysed

Motiv8 was delivered as planned for all four cohorts. Eight participants could form a cohort, the average amount of participants per cohort was seven. A total of 138 individual sessions were delivered during the trial. On average 33.5 sessions were delivered per cohort and varied for each component including on average 22 exercise sessions, 6.5 diet/nutrition sessions, 3 psychology sessions, 1 sleep session, 1 physical health session and 1 pharmacy review. Feedback from facilitators suggested high levels of confidence delivering sessions and the content, duration and frequency was appropriate.

All participants were offered Motiv8. 72.4% (n=21) started the intervention, uptake was lower in the waitlist group (n=11, 64.7%; vs. n=10, 83.3%), and the reasons for not starting were discharge/moving wards (n=6, 75%). For those starting the intervention, almost half attended more than 70% of sessions, one third attended 50-69% of sessions and 19% attended less than 50% of sessions, meeting amber progression criteria. Therefore, before progressing to a full definitive trial we will conduct further work with people with lived experience to identify ways to improve adherence, see (49). Reasons for non-attendance at individual sessions included participant declined (34%), no longer interested (28%), on leave (13%), discharged (11%), COVID (6%), unwell (4%) or sleeping (4%).

### 3.4 Outcomes and estimation

Since the primary aim was to establish feasibility, it was not sufficiently powered to reliably detect significant differences between groups, and secondary analyses are being conducted to explore any potential outcomes of promise for a definitive trial. See **Supplementary Data Sheet 1** for some of the main clinical outcomes of interest.

Focused on informing future economic evaluation, two measures of health status were collected to estimate utility to calculate quality-adjusted life years (QALYS); a generic measure (EQ-5D-5L) and a mental health measure (ReQoL). Complete EQ-5D-5L data was available for 81% (baseline), 84% (10-weeks) and 58% (3-months). Utility could be estimated using the EQ-5D-5L for 55% of participants at all time-points. The mean EQ-5D-5L value at baseline was 0.732 (SD 0.243). As expected, this is lower than population norms (0.893, 35-44) (32). Estimating utility from ReQoL data uses a selection of the items available. Complete ReQoL-UI data was available for 68% (baseline), 84% (10-weeks) and 58% (3-months). Utility could be estimated using the ReQoL for 45% of participants at all time-points. The mean ReQoL-UI value at baseline was 0.846 (SD 0.146). Comparing the EQ-5D-5L and ReQoL derived utilities for participants with complete data for both at baseline, there is a notable difference (EQ-5D 0.767/ReQoL-UI 0.852). This aligns with findings from a larger study of people with schizophrenia (33). Therefore, further work is needed to validate the ReQoL-UI prior to a definitive trial.

### 3.5 Harms

Safety was assessed through tracking incidents and adverse events. Six adverse events occurred. This included participant injury/illness (n=3), one of which resulted in involuntary hospitalisation and two incidents of self-harm (n=2). An unprecedented incident occurred for the latter two cohorts, which resulted in rapid patient discharges and a high staff turnover. However, no adverse events or incidents were related to participation in the trial.

## 4 Discussion

To our knowledge this is the first study of its kind to explore a multidisciplinary lifestyle intervention for adults on forensic inpatient units under randomised conditions. We provide evidence to suggest it is feasible and acceptable to conduct a rigorous, methodologically robust study comparing Motiv8+TAU with TAU. The trial met, (or almost met) all progression criteria including recruitment and randomisation to target and had acceptable retention levels and intervention uptake (49). Blinded conditions were maintained, and no safety concerns raised. This is despite challenging circumstances in which the study was delivered during the COVID-19 pandemic. The trial was not powered to reliably detect any significant differences between clinical outcomes; however, high levels of completion (generally above 90% for people retained and engaged in the study) suggest they are appropriate and acceptable for this population. Feedback from participants was positive and many benefits were reported after taking part (full qualitative and quantitative results are reported elsewhere for completeness).

### 4.1 Clinical implications

Our work addresses several policy guidelines, including the WHO recommendations to manage physical health of people with SMI using lifestyle interventions, and the recent top ten priorities put forward by NHSE to improve the physical health of people with SMI (7, 50). Lifestyle interventions such as Motiv8 are non-invasive and non-stigmatising approaches to healthcare which may prevent the onset of comorbid physical health conditions and reduce the significant loss of life experienced by people with SMI. Our work meets standards from NHSE ‘Managing a healthy weight in adult secure services practice guidance’ which recommend service users should be supported to maintain a healthy weight by accessing multidisciplinary interventions, education, and support, which include service user involvement (51).

. This adds to previous research which has shown that physical health interventions are beneficial, and should form part of standardised care (4{England, 2017 #94)}. Additionally, a person with lived experience of forensic services co-delivered the intervention and received positive feedback. This highlights the benefits of peer support and how this can make a difference to research participants (52, 53). Peer support in forensic care is particularly important and has been found to aid recovery, community reintegration and quality of life. Further developmental work is underway to explore how this can be achieved and implemented in forensic care (54).

The sample was predominately male, and therefore, there is less confidence when suggesting feasibility for females. Previous research has shown distinct differences in clinical presentation, pathways to care, physical health needs and therapeutic approaches for females in forensic services (55–57). The research team experienced difficulties recruiting from female wards including scepticism from clinical teams, and disinterest from the women approached. Further developmental work is underway to establish the appropriateness of Motiv8 for female service users.

Additionally, the inclusion and exclusion criteria were set to allow trial feasibility to be established (e.g. assessing the appropriateness of study processes, written materials, written and verbal assessments and content). This, therefore, resulted in excluding people who did not have sufficient command of English or communication differences which prevented their ability to engage with assessments/group discussions. Forensic services have high rates of neurodiversity including co-morbid autism spectrum disorder and cognitive impairment affecting communication ability (58, 59). Therefore, necessary adaptations are required (such as translated or simplified materials, additional staff support) to allow for more inclusive practice, prior to implementation.

### 4.2 Strengths and limitations

Motiv8 is a valuable contribution to the evidence base which seeks to address the physical health inequalities experienced by people with SMI. It is the first of its kind to be successfully delivered in forensic inpatient services according to a rigorous RCT protocol. The study was delivered as planned and met our original aims, despite challenging circumstances and COVID-19 restrictions. It is a complex multidisciplinary intervention which provides support above and beyond physical health. It was co-produced and co-delivered with significant user-input to ensure acceptability and appropriateness for the patient group, and this peer support was extremely well received. All participants had the chance to engage in the intervention and those who did reported high levels of satisfaction and enjoyment, resulting in immediate real-world impact.

Despite our promising findings, our sample was not wholly representative of the population served as we had a predominately white-male sample; therefore, a definitive trial should attempt to increase inclusivity and diversity. Additionally, there were important mitigating factors which made it difficult to ascertain what was true ‘feasibility’ and what was driven by the unprecedented incident which affected participation, (demonstrated by differences across cohorts). Cohort 1/2 maintained excellent recruitment, retention, and engagement with the intervention. However, Cohort 3/4 had substantially lower retention and attendance and increased loss to follow up. For example, 58.3% retention at 3 months (compared with 82.4% first cohorts) and average attendance was 40.6% of available sessions. Therefore, it is likely rapid discharges contributed to this, and latter cohorts may not represent true feasibility. Nevertheless, as the main reason for participant loss was discharge, it is important that in a definitive trial this factor is mitigated by identifying ways to allow continued participation if they are discharged. Finally, as the main aim of the study was to establish feasibility, the focus here was to present the overall feasibility outcomes of the trial. Therefore, data relating to outcomes and descriptors such as physical health indicators are reported separately to allow a complete and thorough discussion of their implications.

### 4.3 Future research

Given our positive findings, a larger trial is needed to understand the potential benefits and cost-effectiveness of Motiv8. A future trial may also identify potential mechanisms of action and methods of implementation to enhance care provision. Due to the low numbers of female participants, further developmental work is needed with user and clinician input to refine and determine feasibility for female wards. Additionally, further developmental work should be done to account for the impact of the unprecedented incident at the trust, and to ascertain “true” follow-up and attendance rates.

## 5 Conclusion

To conclude, the data provides evidence that the trial is appropriate, feasible and acceptable for patients on forensic inpatient services. Our study provides health providers, commissioners, policy makers, service users and researchers with valuable data regarding evidence-based interventions to enhance physical and mental wellbeing for adults on forensic inpatient services. Further developmental work is needed to create a definitive application to explore the cost-effectiveness and clinical utility of Motiv8 as an adjunct to usual care in NHS services. If Motiv8 is found to result in clinically meaningful changes and prove cost-effective it will have a significant impact on service development, with a view to be incorporated in NICE guidelines.

## Data Availability

The raw data supporting the conclusions of the article are not readily as it contains sensitive information. Requests to access the data should be directed to the corresponding author/s.
