# Supplementary material for: A multidisciplinary weight management intervention for adults with severe mental illness in forensic psychiatric inpatient services (Motiv8): a single blind cluster-randomised wait-list controlled feasibility trial
Source: Front Psychiatry. 2024 Nov 18;15:1457864. doi: 10.3389/fpsyt.2024.1457864 (PMC11609147; doi:10.3389/fpsyt.2024.1457864)
Supplement: Supplementary file 1 [file DataSheet1.docx]

# Supplementary Materials

## Original aims and objectives

| Research Question/Aim(s) | **Objectives** | **Outcomes** |
| --- | --- | --- |
| Primary | To assess acceptability and feasibility of the research trial, associated processes including the intervention, and assessments. | Measured by:   - Recruitment rates - Follow-up retention and questionnaire/outcome response rates - Attendance at sessions - Experience of involvement in the trial - Assessment of safety (SAEs) - Development of a manualised intervention |
| Secondary | Physical Health:  Body composition, blood pressure, cardiovascular fitness, health status  Mental Health:  Wellbeing, depression, anxiety, negative symptoms  Behavioural:  Physical activity, occupational functioning, diet, sleep | Physical Health Measures:   - BMI - BP - Hip/Waist/Chest/Neck circumference - Fitness test   Mental Health Measures:   - WEMWBS - HADS - SNS   Behavioural Measures:   - SIMPAQ - MOHOST - 24 Hr Diet Recall - PROMIS SD Short-Form - PROMIS SRI Short Form   Measures to Support Economic Evaluation:   - EQ-5D-5L - ReQoL - Engagement in care - LUNSERS - Ward activity. |
| Tertiary | Clarify training needs for delivering Motiv8 via a MDT care team, prior to the commencement of a definitive trial. | - Qualitative Interview - Adherence Checklists - Feedback forms and interviews with facilitators - M-BacK Assessment and ESSEN-CES with clinical staff |

Original aims and objectives with corresponding outcome measures.

## Retention Rate According to Cohort

|  |  |  |  |  |
| --- | --- | --- | --- | --- |
|  | **10 week** | | **3 month** | |
|  | n | % | n | % |
| Cohort 1 (n=8) | 8 | 100 | 8 | 100 |
| Cohort 2 (n=9) | 9 | 100 | 6 | 66.67 |
| Cohort 3 (n=4)* | 3 | 75.00 | 3 | 75.00 |
| Cohort 4 (n=8)* | 7 | 87.50 | 4 | 50.00 |
| First two cohorts (n=17) | 17 | 100% | 14 | 82.4% |
| Second two cohorts (n=12) | 10 | 83.3% | 7 | 58.3% |
| **Total Waitlist (n=17)** | **16** | **94.12** | **10** | **58.82** |
| **Total Motiv8 (n=12)** | **11** | **91.67** | **11** | **91.67** |

*affected by incident at trust

## 3. Assessment Completion Based on Participants Engaged and Available to Approach.

| **Assessment Type** | Baseline | | | 10-week | | | 3-month | | |
| --- | --- | --- | --- | --- | --- | --- | --- | --- | --- |
|  | N (approached) | N (complete) | % | N (approached) | N (complete) | % | N (approached) | N (complete) | % |
| **RESEARCH ASSISTANT PACK** | | | | | | | | | |
| Clinical Demographics | 29 | 29 | 100% | 28 | 27 | 96.43% | 23 | 22 | 93.33% |
| Physical Health Measures | 28 | 28 | 100% | 28 | 27 | 96.43% | 21 | 20 | 95.24% |
| Six-Minute Walk | 29 | 28 | 100% | 28 | 25 | 89.29% | 18 | 17 | 94.44% |
| Standing Jump | 28 | 27 | 96.43% | 26 | 24 | 96.15% | 16 | 15 | 93.75% |
| SIMPAQ | 29 | 29 | 100% | 28 | 27 | 96.43% | 23 | 22 | 95.65% |
| 24HR Diet Recall | 29 | 29 | 100% | 28 | 27 | 96.43% | 23 | 22 | 95.65% |
|  |  |  |  |  |  |  |  |  |  |
| **SELF REPORT PACK** | | | | | | | | | |
| Smoking | 28 | 28 | 100% | 28 | 27 | 96.43% | 20 | 20 | 95.24% |
| WEMWEBS | 28 | 28 | 100% | 28 | 27 | 96.43% | 20 | 20 | 95.24% |
| HADS | 28 | 28 | 100% | 28 | 27 | 96.43% | 20 | 20 | 95.24% |
| SNS | 28 | 28 | 100% | 28 | 27 | 96.43% | 20 | 20 | 95.24% |
| PROMIS SD SF | 28 | 28 | 100% | 28 | 27 | 96.43% | 20 | 20 | 95.24% |
| PROMIS SRI SF | 28 | 28 | 100% | 28 | 27 | 96.43% | 20 | 20 | 95.24% |
| EQ-5D-5L | 28 | 28 | 100% | 28 | 27 | 96.43% | 20 | 20 | 95.24% |
| ReQol | 28 | 28 | 100% | 28 | 27 | 96.43% | 20 | 20 | 95.24% |
| LUNSERS | 28 | 28 | 100% | 28 | 27 | 96.43% | 20 | 20 | 95.24% |
|  |  |  |  |  |  |  |  |  |  |

Participant completion rate for individual measures at each time point for participants who could be approached and remained engaged in the trial at the time of assessment.

## 4. Additional Demographic Data

| **AGE in Years** | N | Mean | SD | Range |
| --- | --- | --- | --- | --- |
| All Participants | 29 | 36.5 | 9.9 | 20 to 61 |
| TAU+Waitlist Motiv8 | 17 | 39.6 | 10.6 | 25 to 61 |
| Motiv8+TAU | 12 | 32.1 | 7.2 | 20 to 42 |

## 5. Study Flow Chart


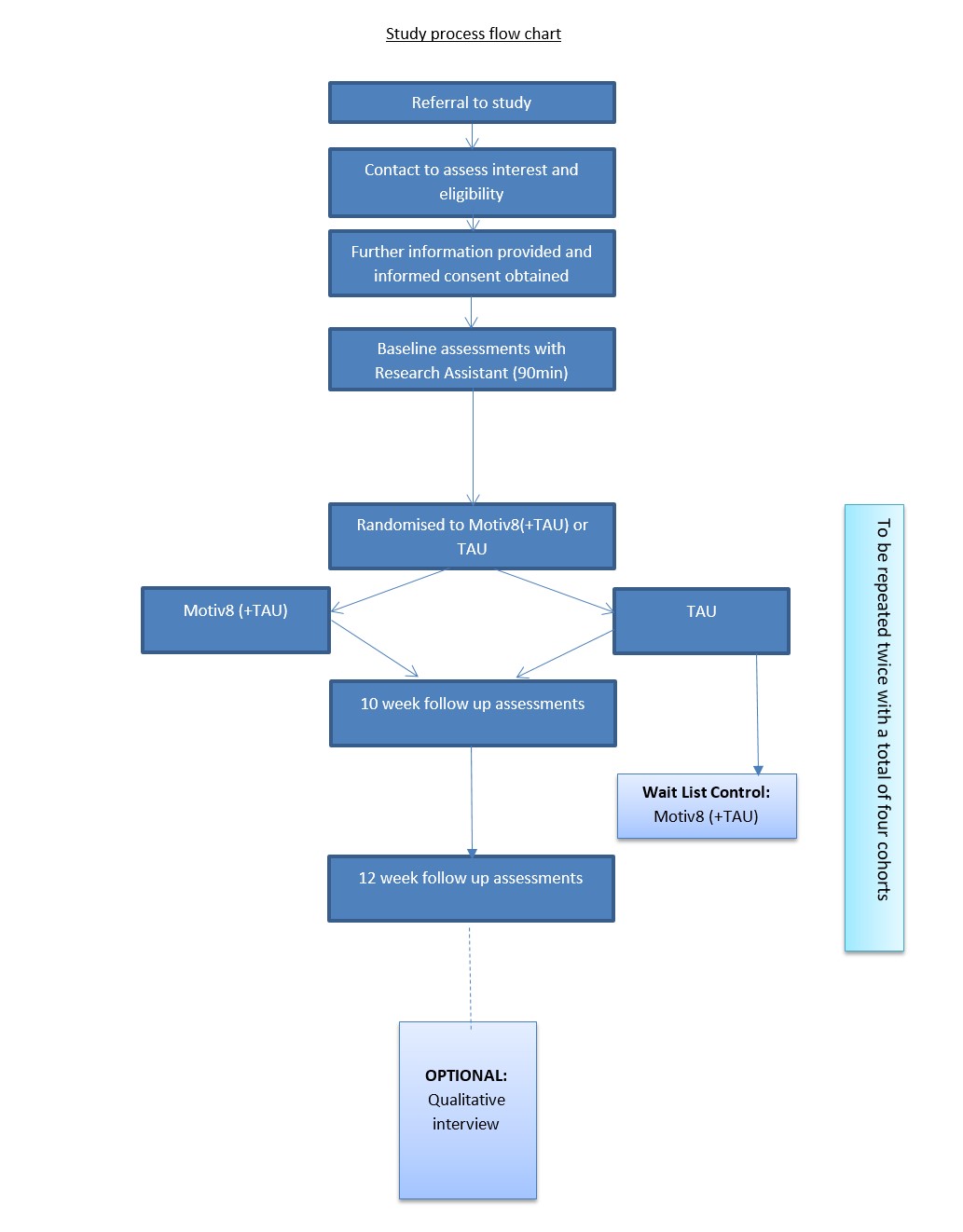


Original flow chart of participant activity before and during the trial.

## 6. Clinical Outcomes of Interest

| Outcome | Trial Arm | Primary Endpoint Comparison | | Post-Motiv8 Comparison  (Waitlist Control Group) | |
| --- | --- | --- | --- | --- | --- |
|  |  | Baseline | Week 10 | Baseline (if data available at month 3) | Month |
| Weight (Kg) | Motiv8 | N = 11  median = 106.3  IQR = (97.2, 143.0) | N = 11  median = 107.4  IQR = (92.7, 141.0) |  |  |
|  | Wait-List Control | N = 16  median = 106.65  IQR = (92.3, 120.8) | N = 16  median = 103.15  IQR = (89.4, 122.0) | N = 11  median = 104.2  IQR = (88.35, 120.2) | N = 12  median = 95.25  IQR = (86.65, 111.7) |
| Waist  Circumference (cm) | Motiv8 | N = 11  median = 127  IQR = (104, 134) | N = 11  median = 128  IQR = (106, 135) |  |  |
|  | Wait-List Control | N = 16  median = 122.75  IQR = (102.25, 132.5) | N = 16  median = 119  IQR = (108.25, 132.25) | N = 11  median = 117.5  IQR = (98, 132) | N = 12  median = 113.5  IQR = (102, 130.25) |
| Hip  Circumference (cm) | Motiv8 | N = 11  median = 120  IQR = (109, 127) | N = 11  median = 119  IQR = (107, 130.5) |  |  |
|  | Wait-List Control | N = 16  median = 110.5  IQR = (105.25, 121.25) | N = 16  median = 111.75  IQR = (103, 123.75) | N = 11  median = 109  IQR = (106.5, 119) | N = 12  median = 106  IQR = (103.5, 121.75) |
| Pulse (bpm) | Motiv8 | N = 11  median = 97  IQR = (86, 103) | N = 11  median = 93  IQR = (81, 100) |  |  |
|  | Wait-List Control | N = 16  median = 79  IQR = (74, 96.5) | N = 16  median = 81.5  IQR = (76.5, 100) | N = 11  median = 77  IQR = (69, 96) | N = 12  median = 93.5  IQR = (87.5, 103) |
| 6-Minute Walk (m) | Motiv8 | N = 11  median = 437.2  IQR = (349.3, 452.4) | N = 10  median = 451.9  IQR = (377.0, 480.0) |  |  |
|  | Wait-List Control | N = 16  median = 415.5  IQR = (387.7, 476.1) | N = 15  median = 435.8  IQR = (405.0, 464.2) | N = 8  median = 413.9  IQR = (391.9, 501.3) | N = 9  median = 499  IQR = (434.2, 531.8) |
| WEMWBS (well-being) | Motiv8 | N = 11  median = 48.5  IQR = (43, 62) | N = 11  median = 49  IQR = (47, 70) |  |  |
|  | Wait-List Control | N = 16  median = 44  IQR = (40, 52) | N = 16  median = 42.5  IQR = (38.5, 49.5) | N = 11  median = 44  IQR = (40.7, 52) | N = 12  median = 45  IQR = (43, 50) |
| HADS Anxiety | Motiv8 | N = 11  median = 5  IQR = (1.2, 8) | N = 11  median = 5  IQR = (2, 11) |  |  |
|  | Wait-List Control | N = 16  median = 6.5  IQR = (5.5, 12) | N = 16  median = 7  IQR = (5.3, 11) | N = 11  median = 6  IQR = (6, 14) | N = 12  median = 9.5  IQR = (4.5, 12.5) |
| HADS Depression | Motiv8 | N = 11  median = 5  IQR = (3.5, 9) | N = 11  median = 5  IQR = (3, 10) |  |  |
|  | Wait-List Control | N = 16  median = 9  IQR = (5, 11) | N = 16  median = 9  IQR = (5.5, 12) | N = 11  median = 6  IQR = (5, 12) | N = 12  median = 8  IQR = (4.5, 10) |

Clinical outcomes of interest for potential primary outcome and secondary outcomes of interest for a definitive trial. Secondary outcomes of interest selected from all outcomes based on previous literature and consultation with people with lived experience.

## 7. Definitions of legal status

| **Legal Status** | **Definition** |
| --- | --- |
| 1983 MHA Section 37 | When a crown or magistrate court deems hospital is a more appropriate place than prison. The Crown Court or Magistrates’ Court issues a hospital order if:   1. A person is charged with, or convicted of, an offence which could lead to a prison sentence. 2. Two medical doctors provide evidence of mental disorder. 3. A court believes a hospital order is most appropriate.   Arrangements are made within 28 days and renewed every 6-months. |
| 1983 MHA Section 37/41 | As above (1983 MHA Section 37) but for people who are deemed a high risk to the public due to the nature of the offence. Additional restrictions added meaning discharge from hospital is only possible if a Secretary of State for Justice agrees. |
| 1983 MHA Section 47 | When a person who is a ‘sentenced prisoner’ (that is having been found guilty of a criminal offence and received a custodial prison sentence), requires transfer to a mental health inpatient hospital for treatment needs which cannot be met in a prison healthcare system. Once mental health is stable the person will be transferred back to prison. In the instance that the sentence duration ends whilst a person is in hospital, they will transfer to a 1983 MHA Section 37. |
| 1983 MHA Section 47/49 | As above (1983 MHA Section 47) with additional restrictions. Movement back to prison, or discharge from hospital is only possible if the Ministry of Justice agree. |
| 1983 MHA Section 3 | When a group of health professionals assess a person’s mental health and decide a patient should be admitted to a hospital and detained there to receive compulsory treatment. |
